# Prognostic significance of the therapeutic targets histone deacetylase 1, 2, 6 and acetylated histone H4 in cutaneous T-cell lymphoma

**DOI:** 10.1111/j.1365-2559.2008.03109.x

**Published:** 2008-09

**Authors:** L Marquard, L M Gjerdrum, Ib J Christensen, P B Jensen, M Sehested, E Ralfkiaer

**Affiliations:** 1 Experimental Pathology Unit, Department of Pathology, Copenhagen Biocentre, Copenhagen University HospitalCopenhagen; 2 Department of Oncology, Copenhagen University HospitalCopenhagen; 3 Finsen Laboratory, Copenhagen Biocentre, Copenhagen University HospitalCopenhagen; 4 Topotarget A/S, CopenhagenDenmark

**Keywords:** acetylation, cutaneous T-cell lymphoma, histone deacetylases, histone H4, immunohistochemistry, survival

## Abstract

Prognostic significance of the therapeutic targets histone deacetylase 1, 2, 6 and acetylated histone H4 in cutaneous T-cell lymphoma

**Aims:**

Aberrant histone acetylation has been associated with malignancy and histone deacetylase (HDAC) inhibitors are currently being investigated in numerous clinical trials. So far, the malignancy most sensitive to HDAC inhibitors has been cutaneous T-cell lymphoma (CTCL). The reason for this sensitivity is unclear and studies on HDAC expression and histone acetylation in CTCL are lacking. The aim of this study was to address this issue.

**Methods and results:**

The immunohistochemical expression of HDAC1, HDAC2, HDAC6, and acetylated H4 was examined in 73 CTCLs and the results related to histological subtypes and overall survival. HDAC1 was most abundantly expressed (*P* < 0.0001), followed by HDAC2; HDAC6 and H4 acetylation were equally expressed. HDAC2 (*P* = 0.001) and H4 acetylation (*P* = 0.03) were significantly more common in aggressive than indolent CTCL subtypes. In contrast, no differences were observed for HDAC1 and HDAC6. In a Cox analysis, elevated HDAC6 was the only parameter showing significant influence on survival (*P* = 0.04).

**Conclusions:**

High expression of HDAC2 and acetylated H4 is more common in aggressive than indolent CTCL. HDAC6 expression is associated with a favorable outcome independent of the subtype.

## Introduction

Cutaneous T-cell lymphomas (CTCL) are a heterogeneous group of non-Hodgkin’s lymphomas defined by clonal proliferation of skin-homing malignant T lymphocytes. CTCL vary from indolent subtypes such as plaque lesions of mycosis fungoides (MF) to more aggressive phenotypes, such as Sézary syndrome (SS) and CD30− peripheral T-cell lymphomas. Survival rates are significantly different between patients with indolent and aggressive phenotypes. Hence, the 5-year survival rate for indolent CTCL is 90%^1^–^3^ compared with a <33% 5-year survival rate for aggressive CTCL.^1^–^3^

Whereas early MF is well controlled by skin-directed therapies or phototherapy,^4^–^9^ patients with more aggressive subtypes require systemic therapy. Interferons, retinoids, single-agent or combined chemotherapy have been used,^10^,^11^ but relapses are frequent^12^,^13^ and most drugs have not improved long-term survival.^10^,^11^,^14^,^15^ New therapies are thus needed for treating CTCL.

Histone deacetylase (HDAC) inhibitors are a promising group of drugs in the treatment of both solid and haematological malignancies. By targeting of HDAC enzymes,^16^ they cause acetylation of core histones and other proteins.^17^ HDAC inhibitors can either be pan-inhibitors that target all 11 isoforms of HDAC enzymes, or selective inhibitors that target specific isoforms or subclasses of HDAC.^18^ HDACs are, together with histone acetyltransferases (HATs), involved in the regulation of transcriptional activity through acetylation and deacetylation of specific lysine residues on core histones. In general, histone acetylation through HAT activity leads to increased transcriptional activity, whereas deacetylation through HDAC activity leads to transcriptional repression.^19^,^20^ HDACs are over-expressed in many malignancies^21^–^25^ and are believed to participate in malignant transformation through transcriptional repression of tumour suppressor genes.^25^–^27^ HDACs normally function together with cofactors that recruit HDACs to target genes^28^ and improper recruitment of HDACs by these cofactors has been identified as a mechanism for the development of malignancy.^29^,^30^ Fusion proteins generated by chromosomal translocations have also shown improper HDAC recruitment, particularly in leukaemia. Although the exact anti-tumour mechanisms of action of HDAC inhibitors are unknown, they have been shown to induce cell cycle arrest, proliferation and apoptosis in neoplastic cells.^31^–^36^

For unknown reasons, CTCL has been the malignant disease with the highest clinical response so far to HDAC inhibitors. Thus, all three small molecule hydroxamates, vorinostat (SAHA), panobinostat (LBH-589) and belinostat (PXD101), as well as the natural product romidepsin (depsipeptide), have demonstrated response rates above 24% in CTCL,^37^ and vorinostat has been approved by the Food and Drug Administration for this indication as Zolinza^TM^ (http://www.fda.gov/bbs/topics/NEWS/2006/NEW01484.html).

Despite this marked sensitivity of CTCL to HDAC inhibitors, studies on HDAC expression in CTCL are lacking. The aim of this study was to improve our understanding of HDAC involvement in the development of CTCL. Thus, we examined the expression of HDAC1, HDAC2 and HDAC6 together with the acetylation of histone H4 in CTCL tissue samples and correlated these results with the histological subtype and clinical outcome.

## Materials and methods

### Patients and tissue samples

Formalin-fixed paraffin-embedded samples from skin lesions from 73 patients diagnosed with primary CTCL during the period 1979–2004 were drawn from the archives of the Department of Pathology, Copenhagen University Hospital. None of the patients participated in clinical trials for HDAC inhibitors. The clinical records were reviewed and all samples were examined by morphology and immunohistochemistry, using as a minimum CD3, CD5, CD4, CD8, CD56 and CD30. Based upon these data, the specimens were reclassified according to the World Health Organization-European Organization for Research and Treatment of Cancer classification and comprised 32 cases of MF with plaque lesions, seven cases of MF with tumour lesions, five cases of MF with transformation to large T-cell lymphoma, four cases of SS, seven cases of CD30+ primary cutaneous anaplastic large cell lymphoma (ALCL), 12 cases of CD30− peripheral T-cell lymphoma (PTL) not otherwise specified (NOS), two cases of extranodal natural killer/T-cell neoplasm, nasal type and four cases classified as precursor plasmacytoid dendritic cell neoplasm. These cases were grouped into two categories, i.e. indolent CTCL, comprising MF plaque, MF tumour and CD30+ large cell lymphoma; and aggressive CTCL, comprising the remaining disease categories.

Information on the clinical outcome could be retrieved in 59 (80.1%) of the cases. The overall survival for these patients ranged from 1 to 360 months, with large variations within each category (Table 1).

**Table 1:** Clinical data from 59 cutaneous T-cell lymphoma (CTCL) patients showing number of patients, range in overall survival and median survival within each CTCL category

Subtype	*N*	OS (months)	Median (months)
Indolent categories			
Mycosis fungoides, plaque stage	27	2–360	117
Mycosis fungoides, tumor stage	6	2–120	61.5
CD30+ c-ALCL*	4	5–120	19.5
Total	37	2–360	84.0
Aggressive categories			
Mycosis fungoides, transformed	5	48–182	60
Sézary syndrome	3	30–240	192
PTL, NOS†	8	5–36	20
NK/T-cell lymphoma	2	1	1
PPDCN‡	4	12–30	25
Total	22	1–240	28.5

* Primary cutaneous anaplastic large cell lymphoma.

† Peripheral T-cell lymphoma, not otherwise specified.

‡ Precursor plasmacytoid dendritic cell neoplasm.

### Control cell lines

The A2780 human ovarian cancer cell line (gift from Dr R. Ozols, Fox Chase Cancer Ctr. Philadelphia, PA, USA) was used as the positive control in the immunohistochemical analyses. Thus, A2780 cells treated with 1 μm belinostat (TopoTarget A/S, Copenhagen, Denmark) for 24 h were used as the positive control for H4 acetylation, whereas untreated A2780 cells were used as negative controls for the absence of H4 acetylation. The P388 murine leukaemia cell line, (gift from F. M. Shabel, SRI, Birmingham, AL, USA) together with a clone of P388 lacking HDAC2 (P388/2C clone 1) was used as the control for HDAC2 expression. Untreated A2780 cells were used as positive controls for reactivity of anti-HDAC1 and anti-HDAC6. Negative controls were performed by substituting the specific antibodies with 2% bovine serum albumin (BSA) in Tris-buffered saline (TBS) (pH 7.6).

### Tests for antibody specificity

Primary antibodies were monoclonal anti-HDAC1 (Upstate, Temecula, CA, USA; 05-614), monoclonal anti-HDAC2 (Abcam, Cambridge, MA, USA; ab12169), polyclonal anti-HDAC6 (Abcam; ab1440) and monoclonal anti-acetylated H4 (clone T25 developed by Ronzoni *et al.*^38^). An initial test for antibody specificity was performed by Western blotting using either whole-cell lysates or histones purified from P388 cells after treatment with 1 μm belinostat for 1 h. Blots were incubated with each of the primary antibodies before development. The appearance of only one band verified antibody specificity. The specificity of each anti-HDAC antibody was further tested in an immunohistochemical absorption assay against the corresponding HDAC peptide (gift from CuraGen Corporation, Branford, CT, USA). The antibody was incubated with excess amount of peptide prior to immunohistochemical analysis. Immunonegativity was then considered to be final proof of antibody specificity.

### Immunohistochemical analysis

Heat-induced epitope retrieval was performed using Tris-hydrochloride (TEG) buffer pH 9 (10 mm Tris-HCl + 0.5 mm ethylene glycol tetraacetic acid) for retrieval of HDAC1, HDAC6, and acetylated H4 or Target Retrieval Solution, Citrate pH 6 (Dako, Glostrup, Denmark; S2369) for retrieval of HDAC2. Peroxidase Blocking Reagent (Dako; S2001) was used for blocking endogen peroxidase activity. Slides were pre-incubated in 2% BSA in TBS (pH 7.6) before primary antibodies were added. Dilution of primary antibodies was: anti-HDAC1 (1:2000), anti-HDAC2 (1:12 000), anti-HDAC6 (1:50) and anti-acetylated H4 (1:13 000). EnVision+ (Dako; K4001/K4003) and diaminobenzidene (DAB)+ (Dako; K3468) were used as the detection system. Slides were counterstained with Mayer’s haematoxylin.

Scoring of immunohistochemistry was based on two parameters: the number of immunopositive tumour cells and their intensity of immunoreactivity. Four different scores were given for the number of positive tumour cells, i.e. 0, <5%; 1, >5% to ≤20%; 2, >20% to ≤50%; and 3, >50% positive cells. Intensity of positivity was given a score of 0 for no positivity, 1 for weak positivity, 2 for moderate positivity and 3 for strong positivity. The sum of the two individual scores defined the final immunoreactivity score of each sample, ending up with seven groups with scores between 0 and 6. Finally, in order to ensure a sufficient number of samples in each group for statistical analysis, the samples were grouped in three expression categories defined as low (score 0–2), moderate (score 3 and 4) or high (score 5 and 6) expression. All reactions were simultaneously scored by two observers (L.M., E.R.) using a double-headed microscope.

### Statistical analyses

The comparison of the probability of expression of HDAC1, HDAC2, HDAC6 and acetylated H4 was done using a repeated measures linear model with ordinal categorical data. Tests for independence between immunohistochemical data and diagnostic groups were done using the chi-squared test with exact probabilities. Spearman rank correlation was calculated as a measure of association between the HDACs and H4 acetylation. Survival was calculated from the day of diagnosis until death or last follow-up. Survival probabilities were estimated by the Kaplan–Meier method and the log rank statistic was used to compare survival curves. Multivariate analysis of protein expression adjusted for indolent/aggressive disease was done using the Cox proportional hazards model. *P*-values <5% were considered to be significant.

### Ethical aspects

This study was approved by the local ethics committee in Copenhagen, Denmark (Journal No. 01 326034) and the Danish Data Protection Agency (Journal No. 2006 41 7116).

## Results

### Expression of HDACs and acetylated H4 in non-lymphoid cells

In addition to immunoreactivity of lymphoid cells (see below), the antibodies against HDAC1, HDAC2 and acetylated H4 showed nuclear labelling of epithelial cells in the epidermis and dermal appendages. The antibody against HDAC6 stained the cytoplasm in these cells and also labelled the cytoplasm in endothelial cells. This reactivity of non-lymphoid cells served as useful internal controls for the immunohistochemical reactions.

### Expression of HDACs and acetylated H4 in CTCL cells

In lymphoid cells, HDAC1, HDAC2 and acetylated H4 were expressed in the nuclei, whereas HDAC6 was mainly cytoplasmic with only weak reactivity of the nuclei in occasional cells. All four antibodies mainly labelled neoplastic cells with atypical nuclei, whereas small, reactive appearing lymphoid cells were negative.

**Figure 1:**
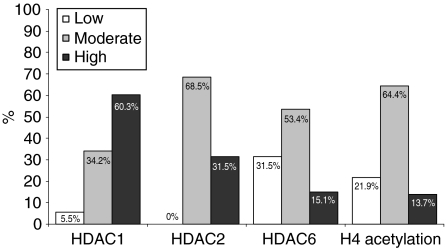
Expression profiles of HDAC1, HDAC2, HDAC6 and H4 acetylation in cutaneous T-cell lymphoma (CTCL) (*n* = 73) showing the percentage of samples in each of three categories of immunoreactivity (low, moderate, high). Significant differences in expression profiles are found between HDAC1 and HDAC2 (*P* < 0.0001) and HDAC2 and HDAC6 (*P* < 0.0001), whereas HDAC6 and acetylated H4 have similar profiles (*P* = 0.36).

**Figure 2:**
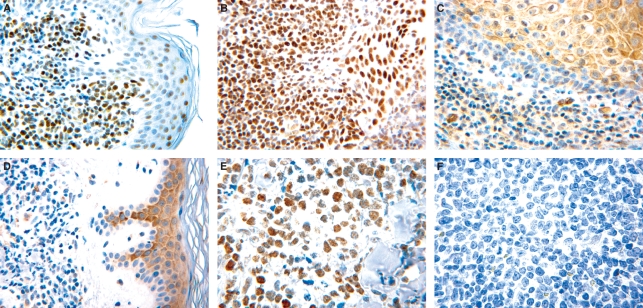
**A**, Mycosis fungoides (MF), plaque stage with high expression of HDAC1 in the nuclei of the lymphoid infiltrate. Note that HDAC1 is also expressed in the nuclei in epithelial cells of the epidermis. **B**, Cutaneous T-cell lymphoma (CTCL), unspecified with high expression of HDAC2 in the nuclei of the neoplastic cells. **C**, MF, tumour stage with high HDAC6 expression in the cytoplasm. Note that HDAC6 is also expressed in the cytoplasm of epithelial cells of the epidermis. **D**, MF, tumour stage, negative of HDAC6 in the lymphoid infiltrate. **E**, CTCL, unspecified with high acetylation of histone H4. Small reactive lymphoid cells are negative. **F**, Precursor plasmacytoid dendritic cell neoplasm negative for H4 acetylation.

Three categories were distinguished based upon the proportion of positive cells and the intensity of immunoreactivity, i.e. low, moderate and high (see Materials and methods). The distribution of all CTCL patients according to these categories is summarized in Figure 1. Representative examples are illustrated in Figure 2.

As shown, HDAC1 was expressed most abundantly, followed by HDAC2 (*P* = 0.002) and HDAC6 (*P* < 0.0001). HDAC6 and acetylated H4 were equally frequently expressed (*P* = 0.36).

### Expression of HDACs and acetylated H4 in CTCL categories

The relationship between immunoreactivity and CTCL categories is summarized in Table 2. Comparisons between indolent and aggressive cases regarding expression of HDAC1 and HDAC6 did not show significant differences (*P* = 0.35 and *P* = 0.89, respectively). In contrast, both HDAC2 (*P* = 0.001) and H4 acetylation (*P* = 0.03) were significantly more common in aggressive than in indolent CTCL. For HDAC2, 55.5% of the aggressive cases showed high expression. Conversely, among indolent CTCL, most cases (82.6%) showed only moderate HDAC2 expression. A similar finding was observed with H4 acetylation, where 22.2% of the aggressive cases showed high expression compared with only 8.7% of the indolent cases. Low H4 acetylation was observed in 30.4% of the indolent cases, whereas only 7.4% of the aggressive cases showed low H4 acetylation. When comparing the expression profiles in patients with indolent and aggressive subtypes, respectively, weak correlations in the expression were observed between all four parameters, i.e. HDAC1, 2, 6, and acetylated H4 (data not shown).

**Table 2:** Expression of HDAC1, HDAC2, HDAC6, and acetylated H4 (H4ace) in different subtypes of cutaneous T-cell lymphoma (CTCL) (*n* = 73). Data show the number and percentage of samples within each group

	HDAC1			HDAC2			HDAC6			H4ace		
Subtype	Low	Moderate	High	Low	Moderate	High	Low	Moderate	High	Low	Moderate	High
Indolent categories												
Mycosis fungoides, plaque stage (*n* = 32)	4	11	17	–	29	3	10	18	4	13	18	1
Mycosis fungoides, tumor stage (*n* = 7)	–	3	4	–	4	3	4	2	1	1	4	2
CD30+ c-ALCL* (*n* = 7)	–	2	5	–	5	2	1	5	1	–	6	1
Total (*n* = 46)	4 8.7%	16 34.8%	26 56.5%	0 0%	38 82.6%	8 17.4%	15 32.6%	25 54.3%	6 13.0%	14 30.4%	28 60.9%	4 8.7%
Aggressive categories												
Mycosis fungoides, transformed (*n* = 5)	–	1	4	–	4	1	–	3	2	–	4	1
Sézary syndrome (*n* = 4)	–	1	3	–	2	2	1	3	–	1	2	1
PTL, NOS† (*n* = 12)	–	4	8	–	4	8	5	4	3	–	9	3
NK/T-cell lymphoma (*n* = 2)	–	1	1	–	–	2	1	1	–	–	2	–
PPDCN‡ (*n* = 4)	–	2	2	–	2	2	1	3	–	1	2	1
Total (*n* = 27)	0 0%	9 33.3%	18 66.7%	0 0%	12 44.4%	15 55.5%	8 29.6%	14 51.9%	5 18.5%	2 7.4%	19 70.4%	6 22.2%

* Primary cutaneous anaplastic large cell lymphoma.

† Peripheral T-cell lymphoma, not otherwise specified.

‡ Precursor plasmacytoid dendritic cell neoplasm.

### Expression of HDACs and acetylated histone H4 in CTCL versus survival

**Figure 3:**
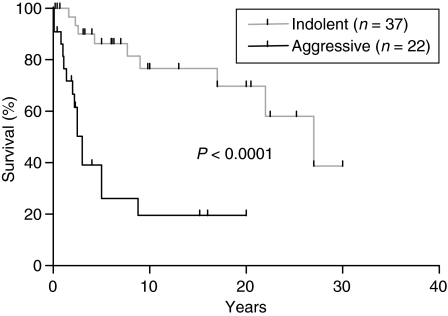
Overall survival of cutaneous T-cell lymphoma (CTCL) patients (*n* = 59) based on indolent versus aggressive subtype. Survival is significantly inferior in aggressive to that in indolent CTCL.

**Figure 4:**
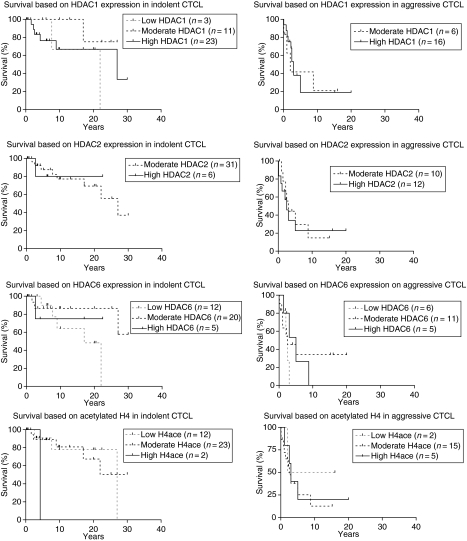
Overall survival of cutaneous T-cell lymphoma (CTCL) patients with either indolent or aggressive subtypes based on expression of HDAC1, HDAC2, HDAC6, or acetylated H4. Cox analysis revealed no significant influence of HDAC1, HDAC2, or H4 acetylation on survival. In contrast, HDAC6 was found to have a significantly beneficial influence on survival.

Overall survival was available for 59 patients. As expected, median survival was significantly different between the indolent and aggressive groups, i.e. 84 months for patients with indolent CTCL compared with 28.5 months for patients with more aggressive disease (*P* < 0.0001). These results are illustrated in Figure 3. To investigate the impact of HDACs and acetylated H4 on survival in indolent and aggressive CTCL we used the Cox-model to adjust for the subtype and examined the influence of negative (score≤ 2) versus positive (score > 2) expression. For HDAC2, we examined the influence of moderate (score≤ 4) versus high (score > 4) expression, due to the fact that no samples showed negative or weak expression. Survival curves are shown in Figure 4. Cox analyses showed no significant influence on survival for HDAC1, HDAC2, or acetylated H4 (see Table 3). In contrast, HDAC6 expression showed a significant beneficial influence on survival [*P* = 0.04, hazard ratio (HR) 0.39, 95% confidence interval 0.16, 0.96] independent of the CTCL subtype.

**Table 3:** Results of Cox analyses showing *P*-values, hazard ratios (HR), and 95% confidence intervals (CI)

Parameter	*P*-value	HR	95% CI
Aggressive	0.0001	7.20	2.66, 19.46
HDAC1	0.30	0.43	0.09, 2.13
Aggressive	0.0003	6.16	2.30, 16.47
HDAC2	0.94	1.03	0.42, 2.55
Aggressive	<0.0001	8.43	3.12, 22.82
HDAC6	0.04	0.39	0.16, 0.96
Aggressive	0.0003	5.82	2.27, 14.93
H4 acetylation	0.55	1.41	0.46, 4.27

The group of indolent subtypes is used as reference to examine the influence of HDAC1, HDAC2, HDAC6, and acetylated H4 on survival. The parameter ‘aggressive’ demonstrates the negative influence of aggressive cutaneous T-cell lymphoma (CTCL) subtype on survival.

## Discussion

Overexpression and improper recruitment of HDACs, particular HDAC1 and HDAC2, have been reported in different malignancies,^21^–^23^,^25^,^30^,^39^–^41^ emphasizing their role in malignant development. Although the influence of HDAC6 in cancer is not as well investigated as for HDAC1 and HDAC2, HDAC6 has been reported to be up-regulated in oral squamous cell carcinoma and the inhibition of HDAC6 has been reported to induce cytotoxicity in multiple myeloma cells as well as to reduce the amount of Bcr-Abl in leukaemia cells.^42^–^44^ The presumptive role of HDACs in malignant diseases has resulted in widespread development of HDAC inhibitors. For as yet unknown reasons, the malignancy most sensitive to HDAC inhibitors is CTCL.

Several clinical trials have examined the sensitivity of CTCL to HDAC inhibitors. Vorinostat, panobinostat, belinostat and romidepsin have all shown efficacy in CTCL with partial and complete responses. Interestingly, HDAC inhibitors have demonstrated equal efficacy in indolent and advanced stages of CTCL in patients who have failed prior systemic therapies. Thus, vorinostat resulted in a response rate of 20–31% in indolent stages (MF stage < IIB) compared with a response rate of 25–30% in advanced stages (MF stage IIB–IVB). All responses were partial (PR), except for one patient with MF stage IIB, who had a complete response (CR).^45^,^46^ Belinostat has shown efficacy in a phase II clinical trial against recurrent and refractory CTCL with CR in one of two patients with ALCL and PRs in two of eight patients with MF and one of four patients with SS.^47^ Romidepsin has also shown efficacy in clinical trials. Thus, treatment with romidepsin has resulted in an overall response rate of 31%, with PRs reported in SS as well as plaque/patch and tumour stages of MF. CRs have also been reported in patients with SS together with patients with PTL, unspecified, and CD30+ ALCL.^48^–^50^ Finally, panobinostat has demonstrated a response rate of six out of 10 patients with advanced-stage CTCL patients, with two CRs and four PRs.^51^ Although objective responses, i.e. complete or partial, are not reached by all CTCL patients, many still benefit from treatment by achieving stable disease and/or pruritus relief.^45^–^47^

Only few immunohistochemical studies have examined the role of HDACs in cancer development and these have focused mainly on solid tumours. Thus, overexpression of HDAC1 and HDAC2 have been reported in gastric cancer^23^,^39^ and, in addition, HDAC2 has been associated with gastric tumour aggressiveness.^39^ Furthermore, HDAC1, HDAC2 and HDAC3 are up-regulated in colonic tumours compared with adjacent normal mucosa.^24^,^52^,^53^ Up-regulation of HDAC1 and nuclear accumulation of HDAC4 have been reported in primary and hormone refractory prostatic cancer compared with benign prostatic hyperplasia.^21^,^22^,^54^ However, in another study no difference was found in the level of HDAC1 expression between normal and malignant prostatic epithelial cells.^55^

In our study, HDAC1 was more abundantly expressed than HDAC2, HDAC6 and acetylated H4. Acetylated H4 and HDAC6 were less expressed in CTCL. The expression profile of H4 acetylation in our study is similar to previous findings in prostatic cancer.^56^ Further, we found significant differences between the expression profiles of HDAC1 and HDAC2. This is in accordance with previous findings in other types of tumour.^53^,^57^ Our results further emphasize the unique functions of HDAC1 and HDAC2, despite their close sequence similarity.

The influence of HDAC1 and HDAC6 on tumour aggressiveness is controversial. Thus, *HDAC1* gene expression was up-regulated in the indolent germinal centre B-cell subtype of diffuse large B-cell lymphoma compared with the more aggressive activated B-cell and type 3 subtypes.^58^,^59^ In contrast, in solid tumours HDAC1 was correlated with more advanced stages of lung cancer^60^ and HDAC6 showed a directly opposite influence in breast cancer and oral squamous cell carcinoma.^44^,^61^ In our study, we found no evidence for the involvement of HDAC1 or HDAC6 in the development of more aggressive CTCL based on their expression in indolent versus aggressive CTCL subtypes. In contrast, we found more extensive expression of HDAC2 in aggressive than indolent CTCL. This is in accordance with previous findings,^24^,^39^ indicating possible involvement of HDAC2 in aggressive CTCL.

Several of the HDAC inhibitors in clinical trials are hydroxamate pan-inhibitors, e.g. vorinostat, belinostat, and panobinostat,^37^,^37^ whereas others such as MS275, MGCD0103 and romidepsin are subtype specific.^18^ Based on the current results as well as those obtained by Zhu *et al.*^24^ and Song *et al.*,^39^ it may be reasonable to develop an HDAC2-specific inhibitor. However, before any such decision is made, more information is needed on the influence of HDACs in malignancies as well as on the anti-tumour mechanism of HDAC inhibitors and how they act in different tumours.

The influence of H4 acetylation in malignancy is questionable. Gain of function mutations in HAT enzymes have been identified in different types of cancer and could in turn lead to increased histone acetylation.^62^ Furthermore, hyperacetylation of H4 is partly caused by loss of function of HDAC1 in chronic myeloid leukaemia.^63^ Thus, H4 hyperacetylation may be a result and not a cause of cancer development. We found that acetylation of H4 was more pronounced in aggressive than in indolent CTCL. In contrast, malignant progression of oesophageal squamous cell carcinoma has been correlated to H4 hypoacetylation.^64^,^65^ In our study, three PTL, unspecified, showed high H4 acetylation. Interestingly, histone acetylation has been shown to up-regulate the development of T-cell receptor gamma gene recombination at least in thymocytes.^66^ Thus, high H4 acetylation may play a role in lymphoma development.

When comparing the influence of HDAC expression and acetylated H4 on survival, HDAC1 did not show significant results. However, a HR <1 suggests that increased HDAC1 expression correlates with better survival in CTCL patients (see Table 3). This is in accordance with previous findings showing that HDAC1 expression is associated with better survival in breast cancer patients, at least in those with small and well-differentiated tumours.^67^,^68^ We could not demonstrate that HDAC2 had an influence on survival. In contrast, HDAC6 expression had a significant influence on survival, with a HR <1, indicating a favourable influence on survival independent of the CTCL subtype (see Table 3). This result agrees with previous findings showing that HDAC6 expression is correlated with better survival in oestrogen receptor-positive breast cancer patients.^61^,^69^ Even though acetylated H4 does not show a significant influence on survival, a HR >1 indicates a possible negative influence of H4 acetylation on survival (see Table 3). This is in contrast to previous findings showing better prognosis for patients with a high level of acetylated H4.^65^

In conclusion, we have found that overexpression of HDAC6 had a beneficial influence on survival and that this was independent of the CTCL subtype. Further, high expression of HDAC2 and acetylated H4 was more common in aggressive than in indolent CTCL.

## Abbreviations:

ALCL: anaplastic large cell lymphoma
BSA: bovine serum albumin
CR: complete response
CTCL: cutaneous T-cell lymphoma
HAT: histone acetyltransferase
HDAC: histone deacetylase
HR: hazard ratio
MF: mycosis fungoides
NOS: not otherwise specified
PR: partial response
PTL: peripheral T-cell lymphoma
SS: Sézary syndrome
TBS: Tris-buffered saline
